# Toll-Like Receptor-Mediated Recognition of Nucleic Acid Nanoparticles (NANPs) in Human Primary Blood Cells

**DOI:** 10.3390/molecules24061094

**Published:** 2019-03-20

**Authors:** Enping Hong, Justin R. Halman, Ankit Shah, Edward Cedrone, Nguyen Truong, Kirill A. Afonin, Marina A. Dobrovolskaia

**Affiliations:** 1 Nanotechnology Characterization Lab, Frederick National Laboratory for Cancer Research Sponsored by The National Cancer Institute, Frederick, MD 21702, USA; enping@gmail.com (E.H.); ankit.486@gmail.com (A.S.); edward.cedrone@nih.gov (E.C.); 2 Nanoscale Science Program, Department of Chemistry, The University of North Carolina at Charlotte, Charlotte, NC 28223, USA; jhalman@uncc.edu (J.R.H.); ntruong8@uncc.edu (N.T.)

**Keywords:** nanoparticles, nucleic acids, NANPs, infusion reaction, interferon, immunotoxicity, Toll-like receptors

## Abstract

Infusion reactions (IRs) create a translational hurdle for many novel therapeutics, including those utilizing nanotechnology. Nucleic acid nanoparticles (NANPs) are a novel class of therapeutics prepared by rational design of relatively short oligonucleotides to self-assemble into various programmable geometric shapes. While cytokine storm, a common type of IR, has halted clinical development of several therapeutic oligonucleotides, NANP technologies hold tremendous potential to bring these reactions under control by tuning the particle’s physicochemical properties to the desired type and magnitude of the immune response. Recently, we reported the very first comprehensive study of the structure–activity relationship between NANPs’ shape, size, composition, and their immunorecognition in human cells, and identified the phagolysosomal pathway as the major route for the NANPs’ uptake and subsequent immunostimulation. Here, we explore the molecular mechanism of NANPs’ recognition by primary immune cells, and particularly the contributing role of the Toll-like receptors. Our current study expands the understanding of the immune recognition of engineered nucleic acid-based therapeutics and contributes to the improvement of the nanomedicine safety profile.

## 1. Introduction

Infusion reactions (IRs) are common adverse effects of a variety of drug products. The underlying causes are incompletely understood and involve many mechanisms. The common and best understood mechanisms of IRs to nanotechnology-formulated products are complement activation-related pseudoallergy (CARPA) and cytokine storm syndrome (CSS). These adverse effects require timely and accurate assessment and intervention. When left unaddressed, IRs may be fatal [1]. Excessive production of cytokines alters the regulation of inflammation and may lead to systemic response and organ damage. The clinical manifestations of CSS include erythematous or purpuric rash, tachypnea, generalized swelling, hypotension, fever, altered mental status, diffuse lymphadenopathy, malaise, tachycardia, and enlargement of liver and spleen [2].

Although cytokine storm can be triggered by traditional formulations of therapeutic proteins and nucleic acids as well as small molecular drug allergens, certain nanocarriers may exaggerate this toxicity [3]. As such, nanotechnology-formulated proteins and nucleic acids require thorough analysis to minimize these side-effects [4]. Cytokine storm has halted clinical translation of several nanoformulations designed for the delivery of nucleic acid therapeutics. For example, MRX34, a lipid nanocarrier-formulated miRNA miR-34, induced severe side-effects and led to the death of several patients [5]. The mechanism underlying cytokine storm may vary depending on the composition and structure of a drug product. In the field of traditional oligonucleotide-based therapies, the recognition of these products by the Toll-like receptors (TLR) is the common reason for the cytokine response [6,7]. While in general, endosomal receptors TLR3, TLR7, TLR8, and TLR9 are recognized as the endosomal sensors of therapeutic nucleic acids, the activation of one or another receptor depends on the type and sequence of the oligonucleotide used as a drug product [6,7].

Nucleic acid nanoparticles (NANPs) have recently evolved as an innovative type of therapeutic in which RNA and/or DNA strands serve as the basis for constructing novel nanomaterials, generating limitless possibilities of novel bottom-up nanoarchitectures [8,9,10,11,12,13,14,15,16]. Unlike other nanocarriers [17], NANPs have unique properties which create a niche for these materials in the current biomedical field. For example, NANP technologies use general knowledge of the structures and biological functions of various natural and artificial classes of RNAs (or DNAs) to tackle specific biochemical problems. These novel assemblies have been extensively characterized in vitro (e.g., by electrophoretic mobility shift assay (EMSA) [18], cryogenic electron microscopy (cryo-EM) [19,20], atomic force microscopy (AFM) [21,22,23], and dynamic light scattering (DLS) [23,24] and demonstrated to effectively operate in vivo [20,25,26,27,28,29,30,31,32]. However, the immunorecognition of novel NANPs is widely unknown and can preclude their further biomedical applications.

Earlier we reported that human immune cells recognize NANPs in a manner similar to the recognition of viruses in that they produce type I interferons (IFNs) [33]. IFNs are common biomarkers in response to viral and bacterial nucleic acids, as well as traditional therapeutic nucleic acids (TNAs) [34,35,36]. Unlike traditional TNAs, we discovered that NANPs are invisible to the immune cells unless delivered inside the cell using lipofection [33]. In our earlier study, we also demonstrated that the physicochemical properties of NANPs determine their recognition by immune cells as well as the magnitude of IFN response produced. Using an oligonucleotide-based inhibitor which blocks all endosomal TLRs, we demonstrated that these receptors are involved in the NANPs’ recognition [33]. However, the specificity of individual TLRs to DNA and RNA nanoparticles with different structures is still unknown. Herein, we report a mechanistic study aimed at identifying the contributions of individual endosomal TLRs in the IFN response to NANPs in human peripheral blood mononuclear cells (PBMC), widely recognized as the best model to study cytokine storm during preclinical characterization of novel drugs [37,38,39,40,41].

## 2. Results and Discussion

### 2.1. Plasmacytoid Dendritic Cells as Main Responders to NANPs in Human PBMC

**Figure 1:**
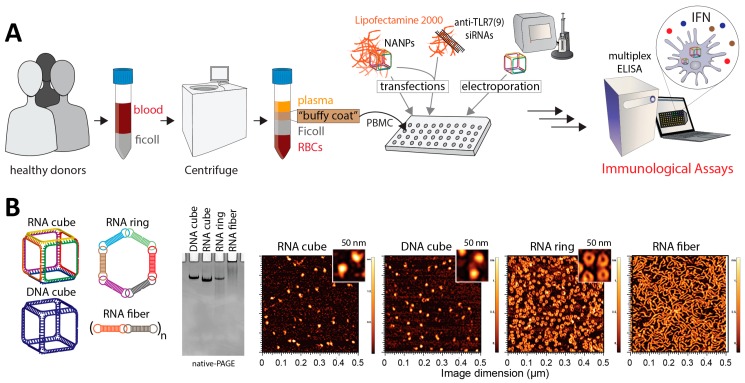
Schematic design of the current study and experimental verification of nucleic acid nanoparticles’ (NANPs’) assemblies. (**A**) Blood from healthy donors was used as a source of peripheral blood mononuclear cells (PBMC), then treated with NANPs with and without prior exposure to the Toll-like receptor (TLR)-inhibiting siRNAs. NANPs were delivered into the cells either by lipofection or electroporation. Type I interferon (IFN) secretion was measured in the culture supernatants by enzyme-linked immunosorbent assay (ELISA). (**B**) RNA cubes, DNA cubes, RNA rings, and RNA fibers were used as model NANPs. All NANPs’ assemblies were confirmed by ethidium bromide total staining native-PAGE and AFM.

Based on our earlier findings [21,22,23,33,42,43] four of the most representative NANPs were chosen as a model system for this study. We chose a small representative group of NANPs to address the influence of their shape (RNA cubes vs. planar RNA rings vs. RNA fibers), and composition (RNA cube vs. DNA cube). The successful formations of tested NANPs were confirmed by native-PAGE and AFM (Figure 1). Then, NANPs were studied in PBMC cultures derived from the blood of healthy donor volunteers. PBMCs were chosen as a model for this study because they accurately represent the immune response of humans, are more sensitive to cytokine-mediated toxicities than in vivo preclinical studies in non-human primates and rodents, and therefore are often referred to as the best model to identify cytokine-mediated toxicities during translational studies of novel therapeutics [37,38,39,40,41]. All NANPs were delivered into the cell either by lipofection, chosen as the main delivery method, or by electroporation, used as the control method (Figure 1). Lipofection was chosen because it is commonly utilized by other researchers, and therefore is representative and relevant to the field of NANPs translational research. Moreover, we previously demonstrated that lipofection delivers NANPs into the endosomal compartment, where TLRs reside; therefore, it is suitable for the assessment of TLR-mediated responses [33]. Electroporation was chosen for direct NANP delivery into the cytosol, bypassing the endosomal compartments and, therefore, avoiding recognition by TLRs [44].

**Figure 2:**
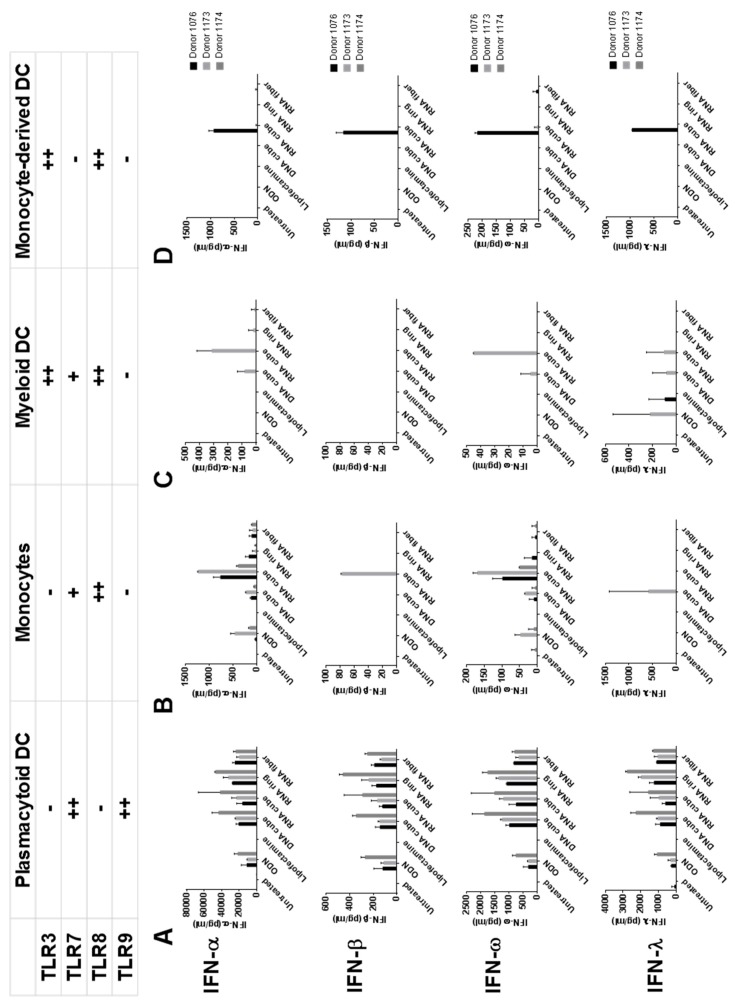
The response of dendritic cell (DC) subsets to delivered NANPs. NANPs were delivered to cells from major DC subsets purified by negative selection, and resulting supernatants were assayed for IFN production. The purified DC subsets tested were (**A**) plasmacytoid DCs, (**B**) monocytes, and (**C**) myeloid DCs. Additionally, isolated monocytes were differentiated into (**D**) monocyte-derived DCs, which were also tested for IFN induction. Some data from individual donors presented in this figure were adapted from our earlier study (1) with permission. ODN = ODN2216, an oligonucleotide, known to induce interferon response and used in our study as a positive control.

To understand the involvement of individual blood cell subtypes in the IFN response to NANPs, we isolated individual cell subtypes from PBMCs and studied IFN stimulation in these cells by model NANPs (DNA cube, RNA cube, RNA ring, and RNA fiber) [21,24,45]. Supernatants from cultures of purified plasmacytoid dendritic cells (pDCs) were found to be the primary responders to all NANPs (Figure 2).

These findings were consistent with our earlier report [33] and in agreement with literature describing the primary role of pDCs in recognition of foreign nucleic acids [6]. Since pDCs express two endosomal TLRs, namely TLR7 and TLR9 [6], and our earlier study in reporter-cell lines suggested the involvement of these TLRs in NANPs recognition [33], we focused on these TLRs for the subsequent detailed analysis.

### 2.2. TLR7 and TLR9 Involved in NANPs Recognition by Human PBMC

By using pan-TLR inhibitory oligonucleotides and a family of TLR-specific reporter-cell lines, we have previously demonstrated that the production of interferons by immune cells requires TLRs [33]. However, the models and design used in our earlier study did not allow for discrimination between individual TLRs. Therefore, to fill the gap in the understanding of NANPs recognition by primary immune cells, we applied siRNA technology to inhibit the expression of TLR7 and TLR9 in primary cells, then challenged these cells with NANPs and assessed the IFN response. Since delivery of siRNA into primary immune cells is a known challenge [44,46,47], we first tested several platforms including but not limited to the lipofection and adeno-associated viral vector delivery of TLR-specific siRNA into the cells. The majority of these approaches were unsuccessful either due to the low delivery efficiency or cell-priming by the vector and delivery vehicles resulting in upregulation of TLRs expression (data not shown). The only approach that showed promising results was the Accell SmartPool siRNA that did not require a special delivery agent and that contained a mixture of three or four pre-defined siRNAs designed against the same target gene.

**Figure 3:**
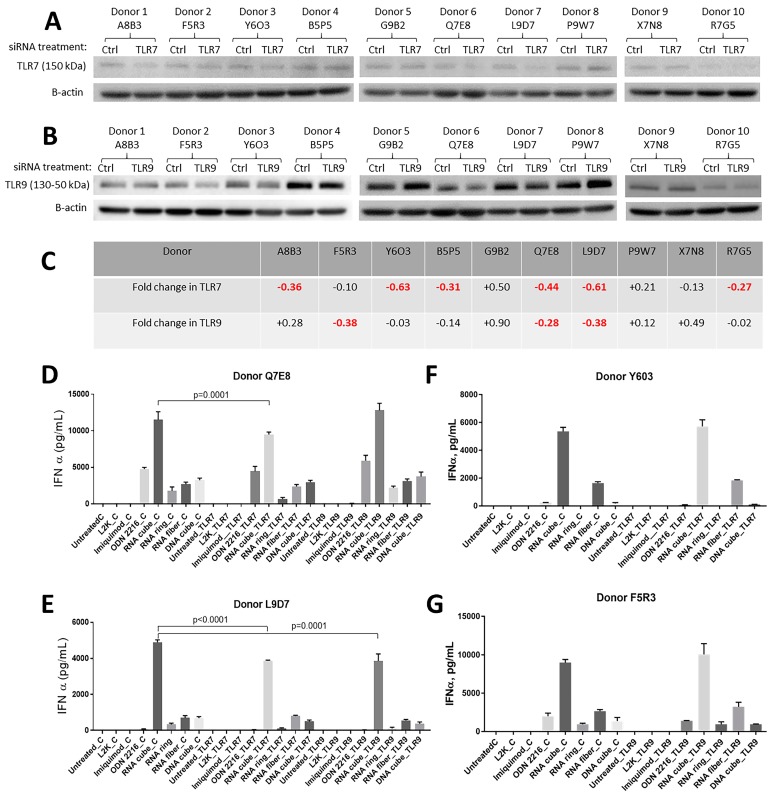
Effects of the inhibition of TLR7 and TLR9 expression on the IFN production by PBMCs treated with NANPs. Freshly isolated PBMCs were either untreated or treated with Accel control siRNA or siRNA specific to either TLR7 or TLR9. NANPs were delivered to cells 36 h after the exposure to siRNA, and the incubation continued for 24 h. At the end of the incubation time, supernatants were collected and analyzed for the presence of IFNα by ELISA, while cell lysates were analyzed for the expression of TLR7 and TLR9 by western blot. (**A**) Selection of donors whose cells responded to Accell SmartPool siRNA by downregulation of the TLR7 protein level. Beta-actin was used to control well loading. (**B**) Selection of donors whose cells responded to Accell siRNA by downregulation of the TLR9 protein level. Beta-actin was used to control well loading. (**C**) Densitometry analysis of western blots shown in A and B. Highlighted in red are the results of the individual donor cells demonstrating at least 25% reduction in TLR7 or TLR9 expression as compared to a respective control group exposed to the control siRNA. (**D**,**E**) Induction of IFNα by NANPs in PBMCs of donor Q7E8 and L9D7 treated with controls and TLR7 or TLR9 siRNA. (**F**) Induction of IFNα by NANPs in PBMCs of donor Y6O3 treated with controls or TLR7 siRNA. (**G**) Induction of IFNα by NANPs in PBMCs of donor F5R3 treated with controls or TLR9 siRNA. A statistically significant difference (*p* < 0.05) is highlighted above bar graphs showing the respective p-value. ODN2216, an oligonucleotide, known to induce interferon response via TLR9 and imiquimod, known to stimulate TLR7, were used in our study as positive controls. L2K is lipofectamine carrier, which was used as a baseline control to normalize for potential carrier-mediated effect.

Due to a known challenge related to the inter-individual variability of primary cells in terms of TLR expression [7,36], we first screened PBMC from 10 individual donors. The cells from randomly selected healthy donors were treated with TLR7 or TLR9 specific Accell SmartPool siRNA, and the expressions of TLR7 and TLR9 were assessed in total cell lysates from these cells by western blot (Figure 3A,B).

Cells treated with control siRNA were used to estimate the efficiency of the inhibition of TLR expression. When bands indicative of TLR7 or TLR9 expression in the siRNA-treated cells from individual donors were compared to that in the cells treated with control siRNA, various degrees of the TLR expression were observed, in that upregulation was detected in some donor cells and downregulation was observed in other donor cells (Figure 3C). Interestingly, cells from the same donor which responded with the highest degree of downregulation of one TLR did not respond equally well with downregulation of another TLR, indicating that the delivery of siRNA into the cell is less likely the reason and suggesting potential inter-individual variability in gene sequence or epigenetic mechanisms of regulation of the expression of individual TLRs in human donors.

Therefore, for the subsequent experiments, we only selected donors whose cells demonstrated at least 0.25-fold (or 25%) decrease in TLR expression (Figure 3C). Donors Y6O3, Q7E8, and L9D7 demonstrating 63%, 44%, and 61 % of TLR7 downregulation by TLR7-specific siRNA, respectively, were chosen as the source of PBMC for the experiment exploring the role of TLR7 in NANPs recognition (Figure 3C). Donors F5R3, Q7E8, and L9D7 demonstrating 38%, 28%, and 38% of downregulation of TLR9 expression by TLR9-specific siRNA, respectively, were chosen as the source of PBMC for the experiment exploring the role of TLR9 in NANPs recognition (Figure 3C).

A statistically significant decrease in IFN secretion induced by RNA cubes and RNA rings in PBMC treated with TLR7-siRNA was observed in cultures from donors Q7E8 and L9D7 (Figure 3D,E). No inhibition of IFN secretion in response to RNA cubes and RNA rings was observed in cells of the donor Y6O3 treated with TLR7-siRNA (Figure 3F). TLR7-siRNA did not affect IFN secretion induced by RNA fibers and DNA cubes in cells from all tested donors (Figure 3D–F). Interestingly, TLR9-siRNA resulted in statistically significant inhibition of IFN secretion in response to RNA cubes in one donor cell culture (Figure 3E). Additionally, TLR9-siRNA inhibited IFN response to RNA rings in cultures from another donor (L9D7, Figure 3E). Although a weak inhibition of IFN secretion in response to DNA cube was noticed in cultures from donors F5R3 and L9D7 pre-treated with TLR9-siRNA, the difference was not statistically significant (Figure 3E,G). Both TLR7- and TLR9-siRNAs inhibited IFN secretion in cultures Y6O3 and F5R3 treated with TLR9-agonist ODN2216 (Figure 3F,G).

### 2.3. Electroporation Suppresses TLR9 Functionality in Human PBMC without Affecting Cell Viability

**Figure 4:**
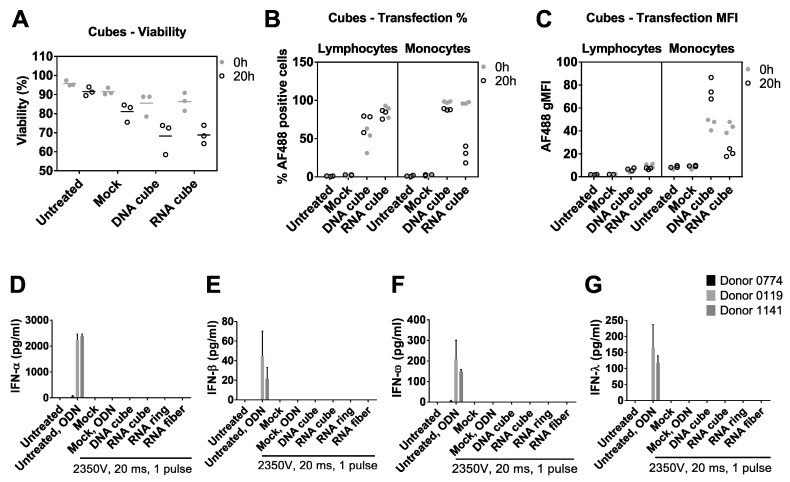
Electroporation of PBMCs with NANPs with a 2350 V, 20 ms pulse. (**A**) Electroporation slightly reduced PBMC viability as measured by acridine orange (AO) and propidium iodide (PI) staining, and resulted in the uptake of AF488-labeled DNA and RNA cubes by both lymphocytes and monocytes, as measured in terms of (**B**) percentage of PBMCs that took up fluorescent nanoparticles, and (**C**) the fluorescence intensity of those cells. PBMC-associated NANPs fluorescence was maintained in PBMCs 20 h after electroporation. In figures (**A**–**C**), each symbol represents data from a single donor. PBMCs electroporated with unlabeled NANPs failed to induce (**D**) IFN-α, (**E**) IFN-β, (**F**) IFN-ω, and (**G**) IFN-λ. Additionally, mock-electroporated PBMCs lost their ability to respond to the positive control, ODN2216 (**D**–**G**).

In order to understand the involvement of non-endosomal signaling in recognition of NANPs, we switched the method of NANP delivery into the cell from lipofection to electroporation [48]. Both the cell viability and transfection efficiency were monitored to select the appropriate electroporation conditions which allowed for concurrent high delivery and viability (Figure 4A–C). We used RNA and DNA cubes as model NANPs to select the conditions (Figure 4A–C), and the complete set of NANPs (DNA cube, RNA cube, RNA ring, and RNA fiber) for subsequent analysis of IFN production.

No production of IFN was observed in response to any of the tested NANPs delivered into the cell by electroporation (Figure 4D–G). Moreover, when the known inducer of IFN response via endosomal TLR9, ODN2216, was added to cultures exposed to the same electroporation conditions but not treated with NANPs, no induction of IFN response was observed (Figure 4D–G). When the ODN2216 was added to the cells which were not subjected to the mock electroporation in the same culture, it resulted in high levels of type I and III IFNs consistent with its expected mechanism of action (Figure 4D–G).

This data clearly demonstrates that electroporation negatively affects the function of endosomal TLRs without affecting cell viability, and therefore cannot be used as a reliable method for studying IFN response to NANPs or ODNs delivered directly into the cytosol. This property was not assessed in the details earlier [48]. Our data suggest that thorough model characterization and the use of appropriate controls are essential for the accurate interpretation of results obtained from studies wherein TNAs are delivered by electroporation. Furthermore, our data highlight the need for other less-invasive delivery methods, necessary to understand NANPs’ recognition after delivery into the cell via pathways bypassing endosomal TLRs.

## 3. Materials and Methods

### 3.1. Reagents

RPMI, PBS (used as a negative control in cell culture experiments), fetal bovine serum, penicillin/streptomycin solution, L-glutamine and Ficoll-Paque Plus were obtained from GE Healthcare Biosciences (Pittsburgh, PA, USA). Lipofectamin 2000 was used as a delivery vehicle and a baseline control, and was purchased from ThermoFisher Scientific (Waltham, MA, USA). Accell SmartPool siRNA (control, TLR7, and TLR9) were purchased from Dharmacon (Lafayette, CO, USA). ODN2216, a TLR9 agonist, and imiquimod, a TLR7 agonist, were used as positive controls for interferon assays, and obtained from Invivogen (San Diego, CA, USA). Interferon multiplex and all-subtype IFNa ELISA kits were from Quansys Biosciences (Logan, UT, USA) and PBL Assay Science (Piscataway, NJ, USA), respectively. All DNAs and fluorescently labeled oligos were purchased from Integrated DNA Technologies (IDTDNA.com). The antibodies used for western blotting were anti-human TLR7 (clone D7, Cell Signaling Technologies, Danvers, MA, USA) and anti-human TLR9 (clone eB72-1665, Thermo Fisher Scientific, Waltham, MA, USA), as well as anti-rat and anti-rabbit HRP-linked secondary antibodies (Cell Signaling Technologies, Danvers, MA, USA).

### 3.2. NANPs Synthesis and Characterization

RNAs entering NANPs’ compositions were synthesized via in vitro run-off transcription (IVT) with home-made T7 RNA polymerase. Prior to IVT, the DNA templates and primers (designed to have T7 promoter sequences) were PCR-amplified (MyTaq, Bioline) and column-purified (Zymo Research). IVT was done at 37 °C for 3.5 h in 300 mM DTT, 400 mM HEPES-KOH, 10 mM spermidine, and 120 mM MgCl_2_. The reaction was stopped by incubation with RQ1 DNase (Promega) for 30 min at 37 °C. Individual RNAs were purified by an 8 M urea polyacrylamide gel (8% acrylamide, 19:1) by extracting gel slices and eluting them into 300 mM NaCl, 1X Tris-borate-EDTA overnight at 4 °C using a shaker. RNAs were added to a 2 Xvolume of 100% ethanol and cooled to –20 °C for 3 h. RNAs were precipitated in 2.5 volumes of 100% ethanol, rinsed with 90% ethanol, vacuum-dried, and dissolved in endotoxin-free water (HyClone). The concentrations of the samples were determined using a NanoDrop2000. NANP compositions used in this project can be found in the Supplementary Materials.

All NANPs were assembled one-pot by mixing the constituent strands at equimolar concentrations (1 μM final) in an assembly buffer (89 mM Tris-borate (1X TB), 2 mM MgCl_2_, 50 mM KCl) (2). Cubes and anticubes were assembled by mixing strands in endotoxin-free water and heating the mixture to 95 °C for two minutes, snap-cooling it to 45 °C, incubating for 2 min, adding 5X assembly buffer and finally incubating at 45 °C for an additional 30 min. Rings, antirings, and fibers were assembled by mixing all strands in endotoxin-free water, heating to 95 °C for 2 min, snap-cooling on ice for 2 min, adding 5X assembly buffer and incubating for 30 min at 30 °C. Following the assembly protocols, all NANPs were confirmed via non-denaturing polyacrylamide gel electrophoresis (run in a cold room at 300 V, 150 mA, for 30 min), native-PAGE (8% acrylamide (37.5:1), 1X TB, 2 mM MgCl_2_), and visualized with a Bio-Rad ChemiDoc MP System using total staining with ethidium bromide.

AFM imaging of NANPs was done on a MultiMode AFM Nanoscope IV system (Bruker Instruments, Santa Barbara, CA) in tapping mode and images were collected with a 1.5-Hz scanning rate using a TESPA-300 probe from Bruker (resonance frequency of 320 kHz, spring constant of about 40 N/m) and processed by the FemtoScan Online software package (Advanced Technologies Center, Moscow, Russia) [49,50].

### 3.3. Primary Human Peripheral Blood Mononuclear Cell (PBMC) Isolation and Treatment with NANPs

Blood was obtained under NCI-at-Frederick Protocol OH9-C-N046. The blood was collected from healthy donors and anti-coagulated with Li-heparin. It was mixed 1:1 with PBS and layered onto Ficoll-Paque Plus, then centrifuged at 900 g with low acceleration and no brake. PBMCs at the buffy coat were collected, washed twice with 1X HBSS, then resuspended in complete RPMI medium (RPMI 1640 with 10% FBS, 2 mM L-glutamine, and penicillin/streptomycin). Live cells were enumerated and used in subsequent experiments. To stimulate PBMCs with NANPs for cytokine induction assessment the cells were seeded at 1.25 × 10^6^ cells/mL in 96-well U-bottomed plates, 160 μL per well. Nanoparticles at 1 μM stock solution were complexed to Lipofectamine 2000 at a 5:1 v/v ratio. After 30 min incubation at room temperature, complexed NPs were made up to 50 nM in OptiMEM and added to PBMCs at 40 μL per well, for a final stimulation concentration of 10 nM. After 20 h incubation at 37 °C, supernatants were collected and analyzed for cytokines by multiplexed ELISA. IFN-α samples above the upper quantitation limit were re-assayed using an all-subtype IFN-α ELISA. Isolation and characterization of individual cell subsets were performed as described earlier [18].

### 3.4. Electroporation of PBMCs with Nucleic Acid Nanoparticles.

Nanoparticles were electroporated using the Neon^®^ Transfection System (Invitrogen, Carlsbad, CA) according to manufacturer instructions. Electroporation settings were optimized by mock electroporation of PBMCs and assessing viability by AOPI staining; parameters that yielded > 90% viability were tested further for the electroporation of Alexa Fluor 488-labeled DNA duplexes. The lowest voltage capable of yielding the highest cell viability and DNA transfection was 1X 2350V, 20 ms pulse, which was used for all electroporation studies. Prior to electroporation, PBMCs were resuspended at 10 × 106 cells/mL in electroporation buffer T. 100 μL of cells was then mixed with 10 pmol of nanoparticles and electroporated with a single 2350V, 20 ms pulse. Electroporated cells were then immediately transferred to RPMI medium, supplemented with 10% FBS and 2 mM L-glutamine, but without antibiotics. If any additional stimulation was required (i.e., ODN2216), cells were rested for 1 h before adding reagents. Electroporated PBMCs were cultured at 1 × 106/mL overnight before assaying for viability by AOPI, and collecting supernatants for cytokine assays.

### 3.5. Western Blot Analysis of TLR Expression.

siRNA-treated TLR reporter cells were washed with 1X HBSS and pelleted. The cell pellets were incubated with radioimmunoprecipitation assay (RIPA) buffer (Boston BioProducts, Ashland), supplemented with Halt^TM^ Protease and Phosphatase Inhibitor Cocktail (Thermo Fisher Scientific, Waltham, MA, USA) for 10 min at 4 °C. The partially lysed cells were sonicated for 10 s at 1 amplitude to completely lyse the cellular organelles and centrifuged at 15,000× *g* for 5 min to remove debris. Lysate protein concentrations were measured by a bicinchoninic acid assay (BCA) protein assay kit (Thermo Fisher Scientific, Waltham, MA, USA) and 10 μg protein was loaded on Novex 4–20% Tris-Glycine gels. After gel electrophoresis, samples were transferred on polyvinylidene difluoride (PVDF) membranes overnight. Membranes were blocked in 5% non-fat milk in PBS-T (0.075% Tween 20 in PBS) overnight at 4 °C to reduce nonspecific signals. Blots were probed for appropriate targets using primary and secondary antibodies, and protein bands were visualized using BM Chemiluminescence Western Blotting Substrate (POD) (Roche, Basel, Switzerland). The blot images were acquired using the G:Box Chemi XX9 gel documentation system and GeneSys software from Syngene USA (Frederick, MD, USA). All the images were adjusted for brightness and contrast throughout the blots before band intensity quantification with ImageJ software. Relative changes in target protein were calculated by normalizing the intensity of target protein with β actin as loading control followed by a comparison of treated samples with vehicle control.

### 3.6. siRNA Delivery

Freshly isolated PBMC were seeded in 6-well plates using Accell delivery medium with 1% FBS and Accell control or TLR-specific siRNAs at 1 μM. An equivalent volume of nuclease-free water was used as a negative control. Cells were cultured for 72 h at 37 °C. On day 3, the cells were split so that some of them were used to prepare cell lysate for the subsequent analysis by western blot, while others were treated with NANPs and had their incubation continued for the additional 24 h. After that, the culture supernatants were collected and analyzed for the presence of interferons.

### 3.7. Statistical and Data Analysis

Analyses were performed using GraphPad Prism 7 software (GraphPad Software, La Jolla, CA, USA).

## 4. Summary and Conclusions

We demonstrated for the first time that in human primary blood cells, endosomal TLR7 is involved in the initiation of the interferon response to RNA cubes and rings, but not to RNA fibers nor DNA cubes. The data also suggested a potential role of TLR9 in recognition of RNA cubes. However, a potential cross-reactivity between TLR9-siRNA and TLR7 polyform or a compensatory mechanism between TLR9 and TLR7 in one of the donors whose blood cells generated this response were not ruled out. These findings broaden the current knowledge regarding the role of Toll-like receptors in recognition of non-traditional nucleic acid therapeutics and point out to the importance of the shape and 3D structure as NANPs parameters critical for TLR-mediated interferon response. Furthermore, we demonstrated that electroporation, when used as a method of NANPs delivery into the cells, affects the ability of endosomal TLRs to initiate IFN response without affecting cell viability. This important finding suggests that electroporation cannot be used as a tool for mechanistic studies investigating molecular recognition of NANPs.

## Supplementary Materials

The following are available online at https://www.mdpi.com/1420-3049/24/6/1094/s1, all sequences used in this projects.
