# Erratum: Hong, E., et al. Toll-Like Receptor-Mediated Recognition of Nucleic Acid Nanoparticles (NANPs) in Human Primary Blood Cells. *Molecules* 2019, *24*, 1094

**DOI:** 10.3390/molecules24213852

**Published:** 2019-10-25

**Authors:** Enping Hong, Justin R. Halman, Ankit Shah, Edward Cedrone, Nguyen Truong, Kirill A. Afonin, Marina A. Dobrovolskaia

**Affiliations:** 1 Nanotechnology Characterization Lab, Frederick National Laboratory for Cancer Research Sponsored by The National Cancer Institute, Frederick, MD 21702, USA; enping@gmail.com (E.H.); ankit.486@gmail.com (A.S.); edward.cedrone@nih.gov (E.C.); 2 Nanoscale Science Program, Department of Chemistry, The University of North Carolina at Charlotte, Charlotte, NC 28223, USA; jhalman@uncc.edu (J.R.H.); ntruong8@uncc.edu (N.T.)

The *Molecules* Editorial Office wishes to make the following erratum to this paper [1].

The reference citation in the legend of Figure 2 was incorrect in the published paper in Molecules [1]. Therefore, it is corrected by the Editorial Office from:

(**Figure 2.** The response of dendritic cell (DC) subsets to delivered NANPs. NANPs were delivered to cells from major DC subsets purified by negative selection, and resulting supernatants were assayed for IFN production. The purified DC subsets tested were (**A**) plasmacytoid DCs, (**B**) monocytes, and (**C**) myeloid DCs. Additionally, isolated monocytes were differentiated into (**D**) monocyte-derived DCs, which were also tested for IFN induction. Some data from individual donors presented in this figure were adapted from our earlier study (1) with permission. ODN = ODN2216, an oligonucleotide, known to induce interferon response and used in our study as a positive control.) To:
(**Figure 2.** The response of dendritic cell (DC) subsets to delivered NANPs. NANPs were delivered to cells from major DC subsets purified by negative selection, and resulting supernatants were assayed for IFN production. The purified DC subsets tested were (**A**) plasmacytoid DCs, (**B**) monocytes, and (**C**) myeloid DCs. Additionally, isolated monocytes were differentiated into (**D**) monocyte-derived DCs, which were also tested for IFN induction. Some data from individual donors presented in this figure were adapted from our earlier study [33] with permission. ODN = ODN2216, an oligonucleotide, known to induce interferon response and used in our study as a positive control.)

We apologize for any inconvenience caused to the readers by this mistake. The manuscript will be updated and the original will remain online on the article webpage. 
