# Supplementary material for: Toll-Like Receptor-Mediated Recognition of Nucleic Acid Nanoparticles (NANPs) in Human Primary Blood Cells
Source: Molecules. 2019 Mar 20;24(6):1094. doi: 10.3390/molecules24061094 (PMC6470694; doi:10.3390/molecules24061094)
Supplement: Supplementary file 1 [file molecules-24-01094-s001.pdf]

Supporting Information for

Toll-like Receptor-mediated Recognition of Nucleic Acid Nanoparticles (NANPs) in Human Primary Blood Cells

Enping Hong [1], Justin Halman[2], Ankit Shah[1], Edward Cedrone[1], Nguyen Truong[2], Kirill Afonin[2]*, and Marina A. Dobrovolskaia [1]*

1 Nanotechnology Characterization Lab, Frederick National Laboratory for Cancer Research Sponsored by the National Cancer Institute, Frederick, MD 21702, USA; neunb@mail.nih.gov

2 Nanoscale Science Program, Department of Chemistry, University of North Carolina at Charlotte, Charlotte, NC 28223, USA

* Correspondence: marina@mail.nih.gov ; Tel.: +01-301-228-4937 and kafonin@uncc.edu; Tel.:+01-704-6870685

NANP compositions used in this project (5'→3')

To assemble the NANPs the following strands are mixed at equimolar concentrations, followed by the assembly protocol described in Methods.

RNA cube
rA: GGCAACUUUGAUCCCUCGGUUUAGCGCCGGCCUUUUCUCCCACACUUUCACG
rB: GGGAAAUUUCGUGGUAGGUUUUGUUGCCCGUGUUUCUACGAUUACUUUGGUC
rC: GGACAUUUUCGAGACAGCAUUUUUUCCCGACCUUUGCGGAUUGUAUUUUAGG
rD: GGCGCUUUUGACCUUCUGCUUUAUGUCCCCUAUUUCUUAAUGACUUUUGGCC
rE: GGGAGAUUUAGUCAUUAAGUUUUACAAUCCGCUUUGUAAUCGUAGUUUGUGU
rF: GGGAUCUUUACCUACCACGUUUUGCUGUCUCGUUUGCAGAAGGUCUUUCCGA
rD-AF488: GGCGCUUUUGACCUUCUGCUUUAUGUCCCCUAUUUCUUAAUGACUUUUGGCC-**AF488**

DNA cube
dA: GGCAACTTTGATCCCTCGGTTTAGCGCCGGCCTTTTCTCCCACACTTTCACG
dB: GGGAAATTTCGTGGTAGGTTTTGTTGCCCGTGTTTCTACGATTACTTTGGTC
dC: GGACATTTTCGAGACAGCATTTTTTCCCGACCTTTGCGGATTGTATTTTAGG
dD: GGCGCTTTTGACCTTCTGCTTTATGTCCCCTATTTCTTAATGACTTTTGGCC
dE: GGGAGATTTAGTCATTAAGTTTTACAATCCGCTTTGTAATCGTAGTTTGTGT
dF: GGGATCTTTACCTACCACGTTTTGCTGTCTCGTTTGCAGAAGGTCTTTCCGA
dD-AF488: GGCGCTTTTGACCTTCTGCTTTATGTCCCCTATTTCTTAATGACTTTTGGCC-**AF488**

DNA anticube
anti-dA: CGTGAAAGTGTGGGAGAAAAGGCCGGCGCTAAACCGAGGGATCAAAGTTGCC
anti-dB: GACCAAAGTAATCGTAGAAACACGGGCAACAAAACCTACCACGAAATTTCCC
anti-dC: CCTAAAATACAATCCGCAAAGGTCGGGAAAAAATGCTGTCTCGAAAATGTCC
anti-dD: GGCCAAAAGTCATTAAGAAATAGGGGACATAAAGCAGAAGGTCAAAAGCGCC
anti-dE: ACACAAACTACGATTACAAAGCGGATTGTAAAACTTAATGACTAAATCTCCC
anti-dF: TCGGAAAGACCTTCTGCAAACGAGACAGCAAAACGTGGTAGGTAAAGATCCC
anti-dC-AF488: **AF488**-CCTAAAATACAATCCGCAAAGGTCGGGAAAAAATGCTGTCTCGAAAATGTCC

RNA ring
nrA: GGGAACCGUCCACUGGUUCCCGCUACGAGAGCCUGCCUCGUAGC
nrB: GGGAACCGCAGGCUGGUUCCCGCUACGAGAGAACGCCUCGUAGC
nrC: GGGAACCGCGUUCUGGUUCCCGCUACGAGACGUCUCCUCGUAGC
nrD: GGGAACCGAGACGUGGUUCCCGCUACGAGUCGUGGUCUCGUAGC
nrE: GGGAACCACCACGAGGUUCCCGCUACGAGAACCAUCCUCGUAGC
nrF: GGGAACCGAUGGUUGGUUCCCGCUACGAGAGUGGACCUCGUAGC
nrC-AF488: GGGAACCGCGUUCUGGUUCCCGCUACGAGACGUCUCCUCGUAGC-**AF488**

RNA antiring
anti-nrA: GGGAAAGCUACGAGGCAGGCUCUCGUAGCGGGAACCAGUGGACGGUUCCC
anti-nrB: GGGAAAGCUACGAGGCGUUCUCUCGUAGCGGGAACCAGCCUGCGGUUCCC
anti-nrC: GGGAAAGCUACGAGGAGACGUCUCGUAGCGGGAACCAGAACGCGGUUCCC
anti-nrD: GGGAAAGCUACGAGACCACGACUCGUAGCGGGAACCACGUCUCGGUUCCC
anti-nrE: GGGAAAGCUACGAGGAUGGUUCUCGUAGCGGGAACCUCGUGGUGGUUCCC
anti-nrF: GGGAAAGCUACGAGGUCCACUCUCGUAGCGGGAACCAACCAUCGGUUCCC
anti-nrA-AF488: GCUACGAGGCAGGCUCUCGUAGCGGGAACCAGUGGACGGUUCCC-**AF488**

RNA ring/antiring monomers
nrA: GGGAACCGUCCACUGGUUCCCGCUACGAGAGCCUGCCUCGUAGC
anti-nrA: GGGAAAGCUACGAGGCAGGCUCUCGUAGCGGGAACCAGUGGACGGUUCCC

RNA fibers
A: GGGAAUCCAAGGAGGCAGGAUUCCCGUCACAGAAGGAGGCACUGUGAC
B: GGGAACGUAAGCCUCCAACGUUCCCGGAUGCUAAGCCUCCAAGCAUCC
B-AF488: GGGAACGUAAGCCUCCAACGUUCCCGGAUGCUAAGCCUCCAAGCAUCC-**AF488**
